# Supplementary material for: Optimal Time Interval between Neoadjuvant Platinum-Based Chemotherapy and Interval Debulking Surgery in High-Grade Serous Ovarian Cancer
Source: Cancers (Basel). 2023 Jul 6;15(13):3519. doi: 10.3390/cancers15133519 (PMC10340433; doi:10.3390/cancers15133519)
Supplement: Supplementary file 1 [file cancers-15-03519-s001.zip › cancers-2447945-supplementary.pdf]

SUPPLEMENTARY MATERIAL

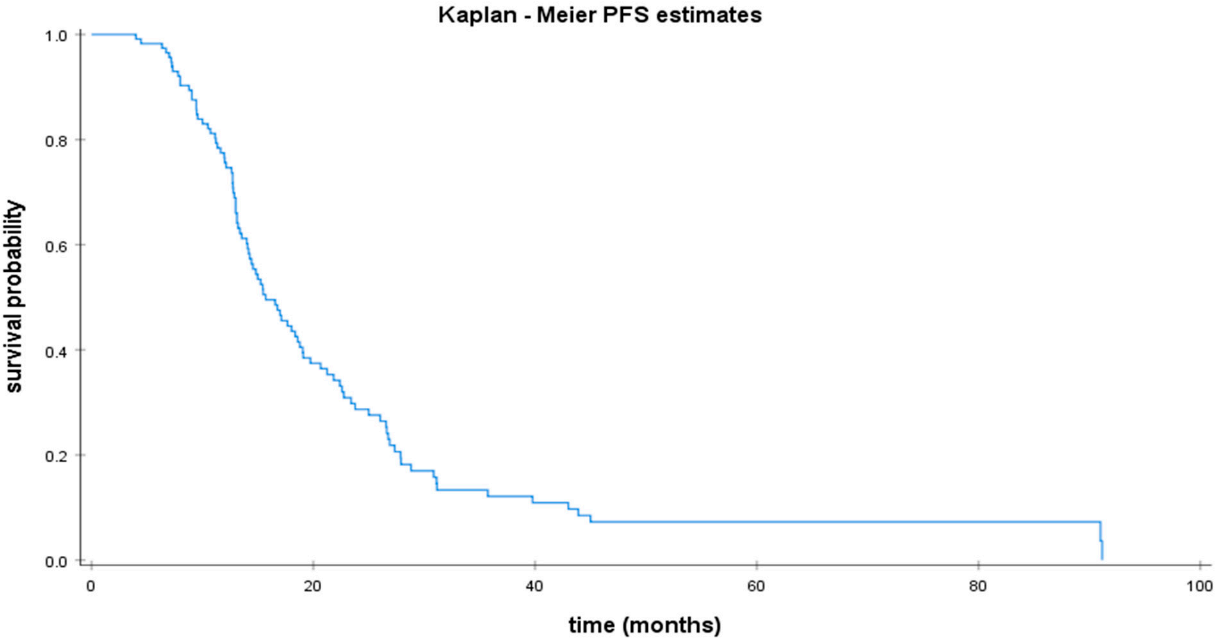

Supplemental Figure S1. Kaplan Meier curve for PFS in the overall population

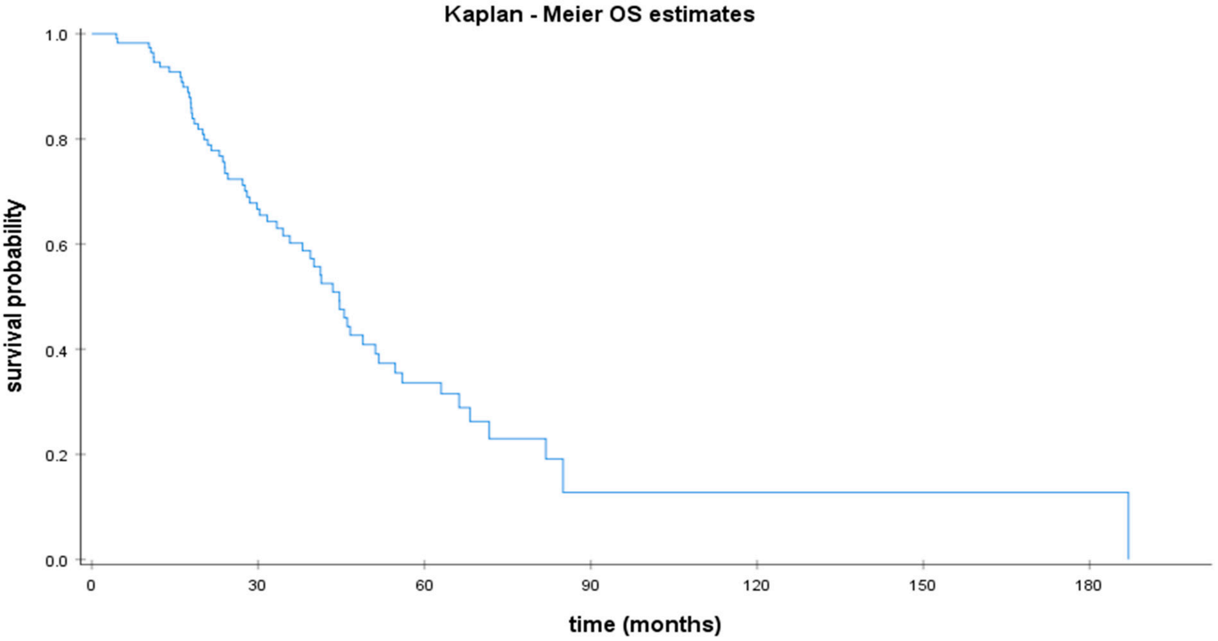

Supplemental Figure S2. Kaplan Meier curve for OS in the overall population
